# Lycorine Derivative LY-55 Inhibits EV71 and CVA16 Replication Through Downregulating Autophagy

**DOI:** 10.3389/fcimb.2019.00277

**Published:** 2019-08-07

**Authors:** Huiqiang Wang, Tingting Guo, Yajun Yang, Lian Yu, Xiandao Pan, Yuhuan Li

**Affiliations:** ^1^CAMS Key Laboratory of Antiviral Drug Research, Institute of Medicinal Biotechnology, Peking Union Medical College, Chinese Academy of Medical Sciences, Beijing, China; ^2^Beijing Key Laboratory of Antimicrobial Agents, Institute of Medicinal Biotechnology, Peking Union Medical College, Chinese Academy of Medical Sciences, Beijing, China; ^3^Department of Pharmacy, Jiamusi University, Jiamusi, China; ^4^Institute of Materia Medica, Peking Union Medical College, Chinese Academy of Medical Sciences, Beijing, China

**Keywords:** enterovirus 71 (EV71), coxsackievirus A 16 (CVA16), lycorine, LY-55, antiviral activity, autophagy

## Abstract

Hand, foot, and mouth disease (HFMD) is a global health concern, especially in the Asia-Pacific region. HFMD caused by Enterovirus 71 (EV71) and Coxsackievirus A16 (CVA16) infection is usually self-limited but occasionally leads to severe pulmonary edema, neurological complications, and even death. Unfortunately, no effective drugs are currently available in clinical practice for the prevention and treatment of HFMD. Thus, anti-HFMD drugs must be urgently developed. A previous study had reported that lycorine could inhibit EV71 replication. In the present study, we found that LY-55, a lycorine derivative, inhibited the replication of EV71 and CVA16 *in vitro* and provided partial protection to mice from EV71 infection, as indicated by the decreased viral load and protein expression levels in muscles, clinical scores, and increased survival rates of infected mice. Mechanistically, LY-55 was not directly viricidal. Instead, the LY-55-mediated inhibition of EV71 and CVA16 was found to be mechanistically possible, at least in part, through downregulating autophagy, which plays an important role for EV71 and CVA16 replication. These findings suggest that LY-55 could be a potential lead or supplement for the development of anti-HFMD agents in the future.

## Introduction

Human enterovirus 71 (EV71) and Coxsackievirus A16 (CVA16), as single-stranded positive-sense RNA viruses belonging to the enterovirus genus of the *Picornaviridae* family, are the two major causative agents of hand, foot, and mouth disease (HFMD) (Wang et al., 2018; Xu et al., 2018). Clinically, infections with the two viruses are self-limited and are often partly associated with neurological diseases, including aseptic meningitis, brain stem encephalitis, and acute flaccid paralysis (Wang et al., 2012, 2017). HFMD has become a serious public health problem, with outbreaks occurring periodically throughout the world, especially in the Asia-Pacific region, including Japan, Malaysia, Singapore, Vietnam, and Mainland China (World Health Organization, 2018). According to the official report of the Chinese Center for Disease Control and Prevention (China CDC), about 2 million children in China develop HFMD every year, among which more than 100 people die (China Center For Disease Control And Prevention, 2017). Therefore, HFMD is a very serious third-class infectious disease in China. However, no effective antiviral drugs are currently available in the clinic to treat HFMD. Thus, developing anti-HFMD drugs is urgently needed.

It takes a long time to screen compounds with anti-EV71 and anti-CVA16 activities, and numerous compounds in various pharmacological medicinal plants have been extensively studied, which not only have potential antiviral activities but also have low cytotoxicity. Polyphenols, terpenoids, alkaloids, and flavonoids are widely distributed natural products with extensive biological and pharmacological activities. Flavonoids, such as formononetin, apigenin and luteolin, inhibit EV71 replication by inhibiting viral RNA replication (Xu et al., 2014; Zhang et al., 2014; Wang et al., 2015). Matrine in alkaloids can inhibit EV71 RNA replication on rhabdomyosarcoma cells (Yang et al., 2012).

**Figure 1:**
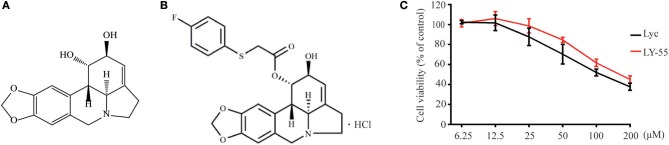
Chemical structure of compounds and CCK assay. **(A)** Chemical structure of lycorine. **(B)** Chemical structure of LY-55. **(C)** CCK assay of compounds.

Lycorine (Figure 1A), an alkaloid component isolated from the bulbs of lycolium, has many biological activities, such as anti-tumor, anti-virus, and anti-inflammation (Kang et al., 2012; Wang et al., 2014; Hu et al., 2015; Ying et al., 2017). Previous studies have shown that lycorine inhibited EV71 replication *in vitro*, and it can significantly improve the symptoms and survival rate of mice infected with EV71 (Liu et al., 2011). Here, we found that the compound LY-55 (2-hydroxy-2,3a^1^,4,5,7,12b-hexahydro-1*H*-[1,3]dioxolo[4,5-*j*]pyrrolo[3,2,1-*de*]phenanthridin-1-yl 2-((4-fluorophenyl)thio)acetate hydrochloride, C_24_H_22_FNO_5_S·HCl, *MW* 491.96, Figure 1B), a lycorine derivative with a therapeutic index superior to that of lycorine, has good activity against EV71 and CVA16 *in vitro*. In the present study, we investigated the antiviral effect of LY-55 *in vivo* and evaluated its antiviral mechanism.

## Materials and Methods

### Cells and Virus

African green monkey kidney (Vero) cells (CCL-81) were purchased from the American Type Culture Collection (ATCC) and cultured in Modified Eagle's Medium (Invitrogen, Carlsbad, CA, USA) supplemented with 10% inactivated fetal bovine serum (FBS; Invitrogen) and 1% penicillin–streptomycin (Invitrogen).

EV71 strain BrCr (VR-1775) and H (VR-1432) were purchased from ATCC. CVA16 strain (shzh05-1/GD/CHN/2005) and EV71 strain SZ98 were kindly provided by Dr. Qi Jin, Institute of Pathogen Biology, Chinese Academy of Medical Science and Peking Union Medical School, Beijing, China. EV71 strain JS-52 was a kind gift from Dr. Xiangzhong Ye, Beijing Wantai Biological Pharmacy Enterprise Co., Ltd. The mouse-adapted EV71 strain (EV71-H-MA) was obtained by the adaptive transmission of mice in our laboratory. All virus strains were all passaged in Vero cells.

### Compounds

LY-55 was synthesized in the Medicinal Chemistry Laboratory of Institute of Materia Medica, Chinese Academy of Medical Sciences, with purity over 98.0%, which met the requirements of Journal of Medicinal Chemistry (Journal of Medicinal Chemistry, 2019). The compound structure was confirmed with ^1^H-NMR and MS spectra. Lycorine hydrochloride (Lyc, MedChemExpress, NJ, USA) was used as reference compounds. Twenty millimeter stock solutions of LY-55 and lycorine were prepared in dimethyl sulfoxide (DMSO). Five millimeter stock solutions of 3-Methyladenine (3-MA, MedChemExpress, NJ, USA) were prepared in DMSO. All compounds were diluted to final working solutions as indicated in experiments.

### Mice

ICR mice, obtained from Beijing Vital River Laboratory Animal Technology Co., Ltd., were housed under specific pathogen-free conditions in individual ventilated cage. The animals were raised and cared for according to the guidelines of the National Science Council of the Republic of China.

### Cytotoxicity Assay

The cytotoxic effect of compounds were assayed by Cell Counting Kit (CCK) (TransGen Biotech, Beijing, China) (Wang et al., 2017, 2018).

### Cytopathic Effect (CPE) Assays

Vero cells were seeded into 96-well plates at a density of 3.0 × 10^4^ cells per well. After 24 h, cells were washed with PBS and then incubated with virus (100 TCID_50_) in serum-free medium for 1 h at 37°C. Then, the unbound viruses were removed and various concentrations of compounds were supplemented for incubation of another 48 h. The reduction of virus-induced cytopathic effect were recorded, then the 50% inhibition concentrations (IC_50_) of compounds were determined using Reed and Muench method, and the selectivity index (SI) values were calculated as the ratio of TC_50_/IC_50_. In addition, the cells were stained with 0.5% crystal violet in 20% ethanol for 15 min at room temperature and the cells were imaged after rinsed with PBS (Wang et al., 2017).

### Mouse Infection Experiments

Mouse-infection experiments were performed under animal biosafety level 2 conditions, and all animal procedures were conducted according to the standard operating procedures approved by the Institutional Animal Ethics Committee, Institute of Medicinal Biotechnology, Chinese Academy of Medical Sciences and Peking Union Medical College. For the lethal EV71 challenge, 12-day ICR mice were intraperitoneally inoculated with EV71-H-MA. After 1 h of infection, the infected mice were intraperitoneally injected with LY-55 (1.5 mg/kg and 0.75 mg/kg) and lycorine (0.75 mg/kg) once daily for 7 days. The placebo group was injected with the same volume of vehicle (water) as control. The symptoms and survival rates of the infected mice were monitored daily for 2 weeks. Eight mice enrolled in each group were dissected at 5 days post-infection (dpi). The muscle tissues prepared from four of each group were fixed in 10% neutral buffered formalin for pathological and immunohistochemical (IHC) analyses, and the muscle tissues prepared from the other four mice were frozen for Western blot and virus titer assays.

The clinical scores were graded with the previously described standards (Liu et al., 2011). The severity of clinical disease was scored as: 0, healthy; 1, hair scattered; 2, limb-shaking weakness; 3, hind limb paralysis; and 4, moribund or dead.

### Virus Titer Determination in the Infected Mice

The muscles were harvested, weighed, and stored at −80°C. They were homogenized in ice-cold MEM by using Precellys Evolution Super Homogenizer (bertin, France), and the virus titers were determined in Vero cells via the CPE assays. In brief, Vero cells (3 × 10^4^ cells/well) were plated into 96-well culture plates for incubation of 24 h. The medium was then removed, and cells were infected with the supernatant of clarified homogenates in serum-free medium for 1 h at 37°C. Then, the unbound viruses were removed and MEM with 2% FBS was supplemented for incubation for another 72 h. The TCID_50_ was calculated by Reed and Muench method.

### Muscle Histological Assessment and Immunohistochemistry

The muscle tissues obtained from infected mice were fixed in 10% neutral buffered formalin solution. Then, the muscle tissue sections were stained with hematoxylin and eosin (H&E) or antibody against EV71 VP1 (GeneTex, California, USA).

### Real-Time qRT-PCR

Total RNA was isolated from cells using the RNeasy Mini Kit (Qiagen, Germantown, MD, USA) and analyzed with the SuperScript III Platinum SYBR Green One-Step qRT-PCR Kit (Invitrogen). The mRNA expression of EV71 VP1 was detected with sense primer 5′- GATATCCCACATTCGGTGA-3′ and antisense primer 5′- TAGGACACGCTCCATACTCAAG-3′ targeting a conserved region of the VP1 gene. The β-actin mRNA was detected using sense primers 5′- CACCATGTACCCTGGCATC-3′ and antisense primer 5′- ACGGAGTACTTGCGCTCAG−3′ (Wang et al., 2017).

### Western Blot Assay

Total cellular proteins were extracted using M-PER Mammalian Protein Extraction Reagent (Thermo Fisher Scientific, Waltham, MA, USA) containing halt protease inhibitor single-use cocktail (Thermo). The extracted total proteins were denatured by adding 5× sodium dodecyl sulfate—polyacrylamide gel electrophoresis (SDS-PAGE) sample buffer (Thermo), followed by boiling for 5 min at 100°C. Approximately 15 μg proteins was applied for SDS-PAGE (Wang et al., 2017). The primary antibodies used in this study included antibodies against β-actin, p-JNK, JNK, SQSTM1/P62, LC3B (Cell Signaling Technology, Beverly, MA, USA), CVA16 (Millipore, MA, USA) and EV71 VP1 (Abnova, Taipei, China). The goat anti-rabbit and anti-mouse HRP-labeled antibodies were obtained from Cell Signaling Technology.

### Immunofluorescence Assay

Vero cells grown on glass coverslips (Thermo) were infected with virus for 1 h. The infected cells were treated with the indicated concentrations of LY-55 for 24 h and then fixed by 4% paraformaldehyde. The cells were permeabilized in 0.5% Triton X-100 at room temperature for 15 min and blocked in PBS containing 1% BSA for 1 h at room temperature. The cells were then incubated with an EV71 VP1 antibody (Abnova) or CVA16 antibody (Millipore) at a dilution of 1:500 for 2 h at room temperature. After washing three times with PBS, the samples were reacted with goat anti-mouse lgG and PE conjugate (TransGen Biotech) for 1 h at room temperature. After washing with PBS, the nucleus was detected with DAPI (Beyotime Biotechnology, Shanghai, China) and images were taken using a fluorescence microscope (Olympus, IX71, Japan) (Wang et al., 2017).

### Statistics Analysis

Statistical analyses were performed using GraphPad Prism 6.0 software. Results are expressed as mean ±SD. Two groups were compared by student's-test, more groups were compared by two-way ANOVA with Holm-Sidak multiple comparisons test (grouped data). Survival data were analyzed using the Kaplan Meier survival analysis. *P* < 0.05 was considered significant.

## Results

### LY-55 Inhibits the Replication of EV71 and CVA16 *in vitro*

We first determined the cytotoxicity of LY-55 by using CCK assay *in vitro*. The result showed that the maximum nontoxic concentration (TC_0_) of LY-55 and lycorine were both 12.5 μM (Figure 1C). As shown in Table 1, the 50% toxicity concentration (TC_50_) of LY-55 and lycorine was 106.36 and 77.27 μM in Vero cells, respectively.

**Table 1:** The efficiency of LY-55 and lycorine against virus *in vitro*.

	**Lycorine**			**LY-55**		
	**TC_**50**_ (μM)**	**IC_**50**_ (μM)**	**SI**	**TC_**50**_ (μM)**	**IC_**50**_ (μM)**	**SI**
EV71(H)	77.27 ± 8.74	2.04 ± 0.39	37.81	106.36 ± 4.62	1.69 ± 0.25	62.72
EV71(SZ98)	77.27 ± 8.74	5.90 ± 1.31	13.06	106.36 ± 4.62	4.85 ± 0.50	21.84
EV71(JS-52)	77.27 ± 8.74	3.20 ± 0.63	24.09	106.36 ± 4.62	3.20 ± 0.63	33.16
EV71(BrCr)	77.27 ± 8.74	3.20 ± 0.63	24.09	106.36 ± 4.62	2.22 ± 0.44	47.82
CVA16	77.27 ± 8.74	3.20 ± 0.63	24.09	106.36 ± 4.62	5.02 ± 0.52	21.19

*TC_50_, 50% toxicity concentration; IC_50_, 50% cytopathic effect (CPE) inhibition concentrations; SI, selectivity index; SI =TC_50_/IC_50_*.

**Figure 2:**
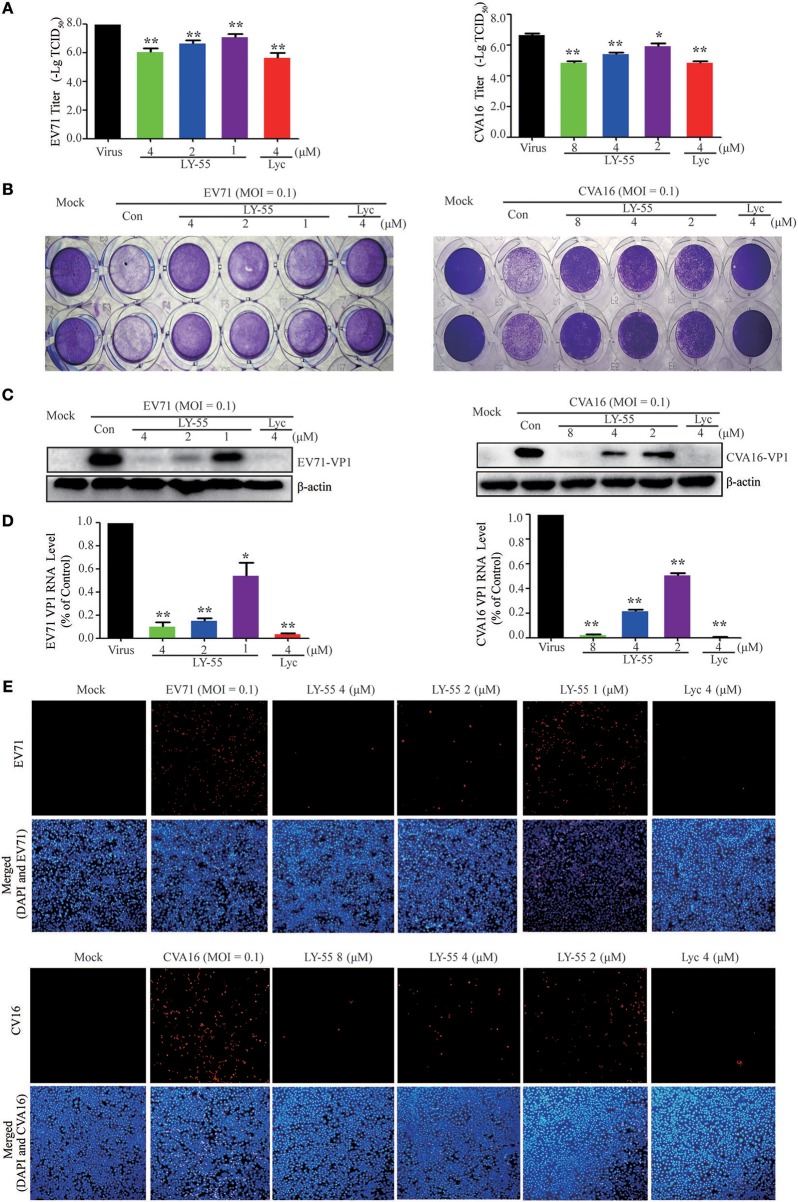
LY-55 inhibited EV71 and CVA16 replication *in vitro*. **(A)** Inhibition of viral titer by LY-55. **(B)** Effects of LY-55 on EV71 or CVA16-induced CPE in Vero cells were determined via crystal violet staining. **(C,D)** Vero cells (9 × 10^5^ cells/well) were plated into 6-well culture plates and infected with EV71 or CVA16 (MOI = 0.1) for 1 h. The infected cells were treated with the indicated concentrations of LY-55 for 24 h. Intracellular viral VP1 protein **(C)** and RNA **(D)** were determined by using Western blot and qRT-PCR assays, respectively. **P < *0.01*, *P < *0.05*. **(E)** EV71 or CVA16-infected cells were revealed by using immunofluorescent detection of VP1 protein by using fluorescence microscopy (×100).

At nontoxic concentrations, LY-55 could decrease the virus titer of EV71 and CVA16 (Figure 2A), and LY-55 could inhibit CPE induced by virus infection in Vero cells, as revealed by crystal violet staining (Figure 2B). As shown in Figures 2C,D, LY-55 treatment decreased viral VP1 protein and RNA levels in a dose-dependent manner *in vitro*. The immunofluorescence assay also revealed that the number of VP1 positive cells was dose-dependently reduced in LY-55-treated cultures (Figure 2E). Those results convincingly demonstrated that LY-55 inhibited EV71 and CVA16 replication *in vitro*.

### LY-55 Was Not Directly Viricidal

**Figure 3:**
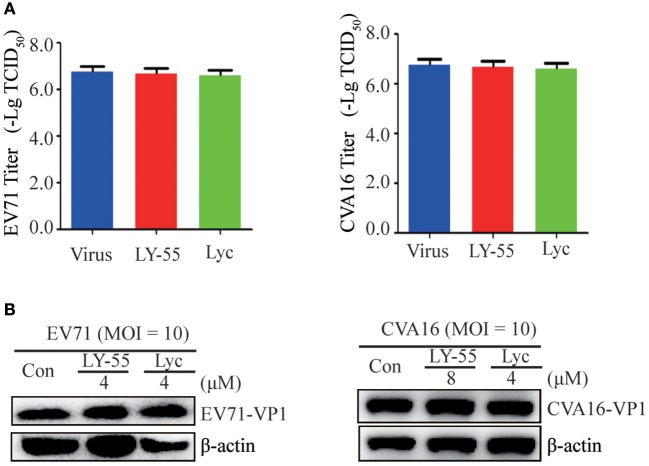
Compound did not inactivate the virus directly. The virus with or without compounds was mixed and incubated at 37°C for 1 h. Vero cells were infected with the virus after centrifugation, and viral loads were detected with titer assay **(A)** and Western blot assay **(B)**.

We first examined whether LY-55 and lycorine directly inactivated the infectivity of EV71 and CVA16 virion particles to investigate their mechanism *in vitro*. By incubating EV71 and CVA16 with LY-55 and Lyc *in vitro* and recovering the virion particles via ultracentrifugation before culture inoculation, we clearly demonstrated via viral titer assay and Western blot assay that virus treatment with LY-55 and lycorine did not significantly reduce its infectivity in Vero cells (Figure 3).

### LY-55 Inhibits Autophagy Induced by EV71 and CVA16 Infection

**Figure 4:**
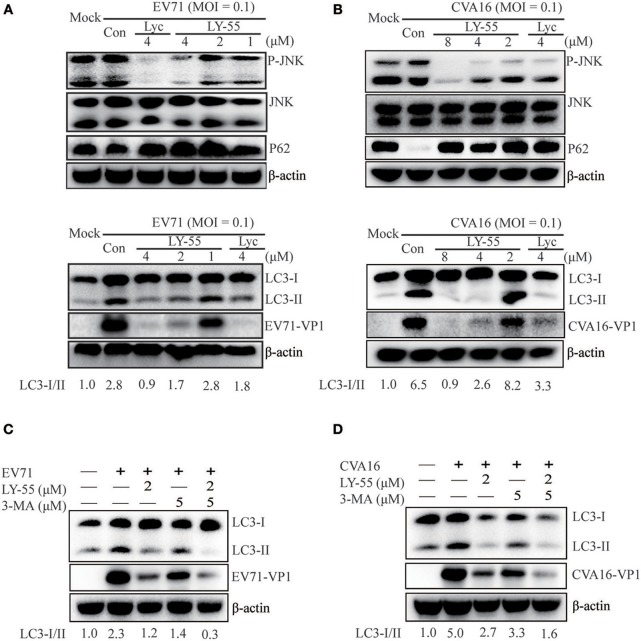
LY-55 could inhibit autophagy induced by EV71 and CVA16 infection. **(A,B)** LY-55 and lycorine can reduce EV71 and CVA16 induced autophagy. Vero cells mock-infected or infected with EV71 or CVA16 (MOI = 0.1) for 1 h. Cells were treated with LY-55 and lycorine for 24 h. Cells were harvested, and the proteins were examined by using Western blot analysis. **(C,D)** LY-55 and 3-MA could synergistically inhibit virus replication through inhibiting autophagy. Vero cells mock-infected or infected with EV71 or CVA16 (MOI = 0.1) for 1 h. Cells were treated with LY-55 and 3-MA for 24 h. Cells were harvested, and the proteins were examined by using Western blot analysis. Sorfware “Gel-Pro analyzer” was used to analysis of the optical density ratio of the bands.

Autophagy induced by virus infection provides support for viral replication. EV71 and CVA16 infection could induce the autophagic machinery to promote virus replication *in vivo* and *in vitro* (Huang et al., 2009; Xi et al., 2013; Shi et al., 2015; Song et al., 2018). The JNK signaling pathway plays an important role in the regulation of cell growth, proliferation, differentiation, migration, and apoptosis. The JNK signaling pathway is closely related to autophagy, and its inhibition could inhibit autophagy (Zhou et al., 2015; Yan et al., 2017; Gao et al., 2018). As shown in Figures 4A,B, LY-55 and Lyc reduced JNK phosphorylation. Accordingly, we found that the lipidated LC3II, a marker for autophagy, decreased in the presence of LY-55 and Lyc. P62 is the most important van protein for selective autophagy, also known as selective autophagy receptor, which serves as a bridge between LC3B and ubiquitinated substrates to be degraded (Boyle and Randow, 2013). P62 binds to ubiquitinized proteins and enters autophagosomes, and finally fuses with lysosomes to form autophagic lysosomes, which are cleared. The content of p62 increased when autophagy was inhibited, and decreased when autophagy was activated. Therefore, autophagy activated by EV71 infection will lead to the degradation of P62 (Xi et al., 2013), while the inhibition of autophagy by LY-55 will inhibit the degradation of P62 and increase its protein level, suggesting that LY-55 was effective in inhibiting autophagy. Therefore, EV71- and CVA16-induced autophagy in Vero cells was attenuated by LY-55 and Lyc. Similarly, 3-MA, an autophagy suppressor (Bao et al., 2018; Slavin et al., 2018), could also inhibit the replication of EV71 and CVA16 (Figures 4C,D). Moreover, LY-55 and 3-MA could synergistically inhibit virus replication through inhibiting autophagy.

### LY-55 Reduced the Mortality of Mice Upon Lethal EV71 Challenge

**Figure 5:**
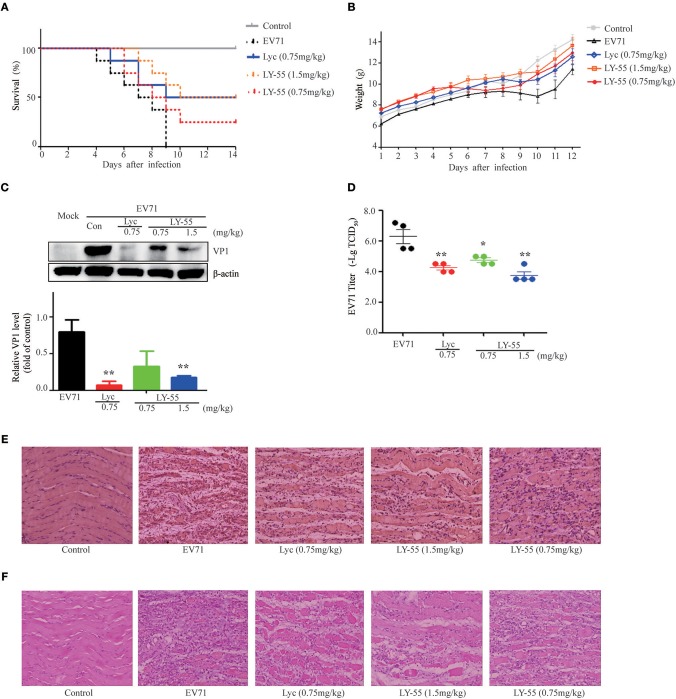
LY-55 treatment reduced the mortality of mice upon lethal EV71 challenge and reduced the EV71 replication post sub-lethal dose EV71 challenge *in vivo*. **(A)** 12-day ICR mice were infected with 10 LD_50_ of EV71-mouse adapted strain (EV71-H-MA), followed by administration of LY-55 and lycorine once a day at the indicated doses. Mice survival was observed for 14 days. **(B–E)** 12-day ICR mice were infected with 1 LD_50_ of EV71-mouse adapted strain (EV71-H-MA), followed by LY-55 and lycorine administration once a day at the indicated doses. Eight mice enrolled in each group were intraperitoneally injected with LY-55 or lycorine for 7 days consecutively and weighed daily for 12 days. **(B)** Eight mice enrolled in each group were dissected at 5 dpi. Muscle tissues prepared from the four mice were used for viral protein expression and viral titer assays. Muscle tissues prepared from another four mice were applied in pathological and IHC analyses. The expression levels of VP1 protein in the muscles were determined by using Western blot analysis at 5 dpi (*n* = 4) **(C)**. Vero cells were infected with a challenge of the diluted muscle homogenates at 5 dpi, and the viral titers were determined by –Log_10_TCID_50_ (*n* = 4) **(D)**. The muscle tissue sections prepared from mice at 5 dpi were stained with EV71 VP1 antibody **(E)** and H&E **(F)**. Each value represents mean ± SD. *P < 0.05, **P < 0.01, vs. EV71-infected mice treated with water.

We utilized the mouse model of lethal EV71 infection to evaluate the effect of LY-55 on inhibiting EV71 infection *in vivo*. The 12-day ICR mice were infected with 10 LD_50_ of EV71-mouse adapted strain (EV71-H-MA). The water-treated control mice succumbed to EV71 infection with mean survival time (MST) of 7.1 ± 2.0 days, and all mice died within 10 dpi (Figure 5A and Table 2). We found that LY-55 and lycorine treatment increased the survival rates of EV71 infected mice. Treatment with 1.5 mg/kg LY-55 provided partial but statistically significant protection from mortality with MST of 11.3±3.1 days (Table 2). However, 0.75 mg/kg LY-55 treatment provided partial but non-statistically significant protection from mortality. Meanwhile, lycorine (0.75 mg/kg) and LY-55 (1.5 mg/kg) treatment protected four out of eight mice from death.

**Table 2:** Impact of treatment with drugs on mortality rates and mean survival time of mice infected with EV71-H-MA.

**Groups**	**Dose (mg·kg^**−1**^)**	**Mortality rate**	**Mean survival time (days)**	**Life extension rate%**
Con	–	0/8	14.0 ± 0.0	–
EV71	–	8/8	7.1 ± 2.0^**^	–
Lyc	0.75	4/8	10.5 ± 3.9^*^^#^	47.9
LY-55	1.5	4/8	11.3 ± 3.1^*^^##^	59.2
LY-55	0.75	6/8	9.3 ± 3.2^**^	31.0

*n = 8; MST value represents mean ± SD; Life extension rate = (MST(Compound)-MST (Virus))/MST(Virus)*;

* *P < 0.05 vs. Con*;

** *P < 0.01 vs. Con*;

# *P < 0.05 vs. EV71*;

## *P < 0.01 vs. EV71*.

### LY-55 Treatment Reduced Viral Replication and Protected Mice From Apparent Symptoms Post Sub-Lethal Dose EV71 Challenge

LY-55 increased mice survival upon lethal EV71 challenge. Next, we also explored the effect of LY-55 against sub-lethal dose EV71 challenge. We found that LY-55 treatment decreased body weight loss (Figure 5B), and EV71 VP1 protein expression level was significantly decreased in infected mice treated with 1.5 mg/kg LY-55 at 5 dpi compared with water-treated control mice infected with the virus (Figure 5C). As expected, treatment with lycorine at 0.75 mg/kg dose markedly reduced EV71 VP1 protein expression at 5 dpi. In addition, LY-55 dose-dependently decreased viral titers in the infected muscles, and treatment with the 1.5 mg/kg LY-55-reduced viral titers by approximately 2 log (Figure 5D). IHC studies demonstrated that the EV71 VP1 protein expression in virus-infected muscles was significantly reduced in 1.5 mg/kg LY-55-treated group at 5 dpi, compared with the infected mice without therapy (Figure 5E). In parallel with the decrease of viral protein expression, LY-55 treatment also improved muscle pathology, as shown in the H&E staining of the muscle tissue sections (Figure 5F). Similar results were obtained in the group treated with lycorine.

**Figure 6:**
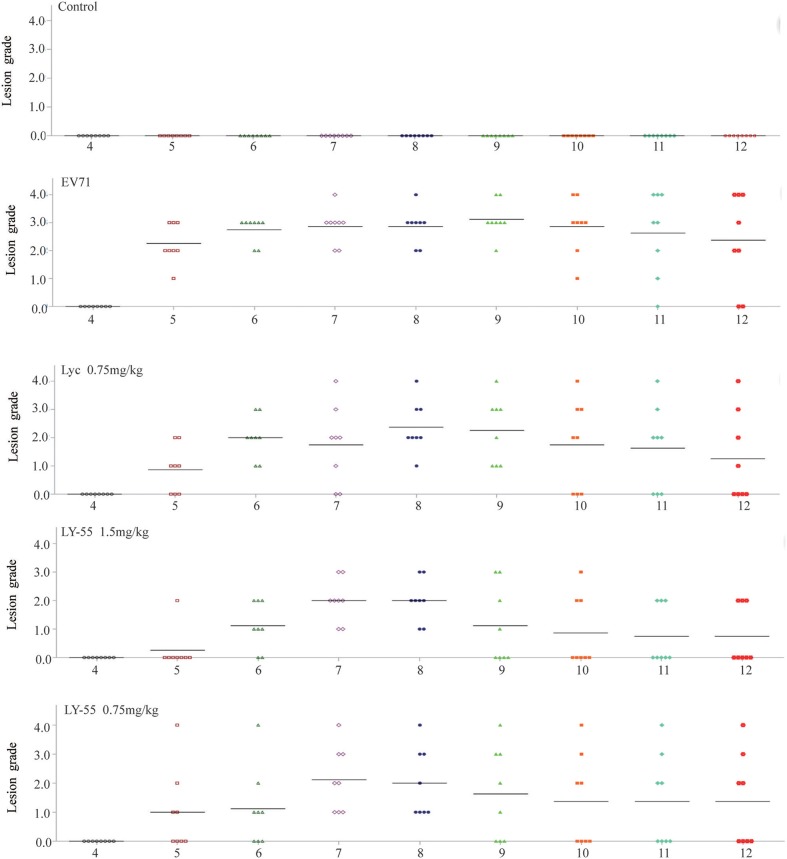
LY-55 treatment protected mice from apparent symptoms post sub-lethal dose EV71 challenge. 12-day ICR mice were infected with 1 LD_50_ of EV71-mouse adapted strain (EV71-H-MA), followed by daily administration of LY-55 and lycorine at the indicated doses. Eight mice enrolled in each group were intraperitoneally injected with LY-55 or lycorine for 7 consecutive days and observed for morbidity daily for 12 days.

The clinical scores of infected mice treated with LY-55 or lycorine were systematically evaluated. Treatment with LY-55 and lycorine delayed the appearance of paralysis compared with that of the vehicle-treated control. The surviving mice in the LY-55 and lycorine group completely recovered within 14 dpi (Figure 6). The water-treated control mice developed transient ruffled hair and paralysis. These results indicated that LY-55 treatment protected the mice from obvious symptoms upon the sub-lethal EV71 challenge.

## Discussion

HFMD is a common and multiple infectious disease, mainly occurring in infants and children under 5 years old, and can be caused by a variety of intestinal viruses (McMinn, 2012; Wang et al., 2018; Xu et al., 2018). EV71 and CVA16 viruses are the two main pathogens causing HFMD. However, no effective antiviral drugs are currently available for the treatment of HFMD. Thus far, numerous chemical compounds or natural products were reported to have good anti-EV71 activity *in vitro*, but most of the antiviral efficacy of these compounds has not been studied *in vivo* (Li et al., 2013; Pu et al., 2013; Wang et al., 2013, 2017). Only a small number of compounds that could inhibit EV71 infection *in vivo* have been reported, among which lycorine was found to have a good anti-EV71 effect *in vivo* (Liu et al., 2011). Here, we reported that LY-55, a lycorine derivative with a therapeutic index superior to that of lycorine, has good activity against EV71 and CVA16 *in vitro*.

Viral infection induces autophagy and virus-induced autophagy also supports viral replication. It has been reported that EV71 infection can induce autophagy to promote EV71 replication *in vivo* and *in vitro* (Huang et al., 2009). Therefore, inhibition of autophagy may be a promising strategy to inhibit the replication of EV71 and CVA16. Our previous studies found that berberine inhibits EV71 replication by downregulating autophagy (Wang et al., 2017). In this study, preliminary mechanism studies showed that LY-55 could effectively inhibit autophagy induced by EV71 and CVA16 infection, which is important for virus replication (Lee et al., 2014; Fu et al., 2015). LY-55 suppressed LC3BII in EV71- and CVA16-infected Vero cells. Given that JNK MAPK is closely related to autophagy, its inhibition could prevent autophagy. LY-55 and lycorine reduced JNK phosphorylation induced by virus infection (Figure 4). Previous studies have shown that lycorine blocks the elongation of the viral polyprotein during translation to inhibit EV71 replication (Liu et al., 2011). Guo et al. found that 1-acetyllycorine suppressed the proliferation of multiple strains of EV71 in various cells through EV71 2A protease targeting (Guo et al., 2016). We found a new antiviral mechanism for LY-55 and lycorine to against EV71 and CVA16 replication, at least partly by inhibiting autophagy. Our findings supplement the antiviral mechanism of lycorine and its derivatives and provide a new basis for elucidation of the antiviral mechanism of lycorine and its derivatives.

The antiviral effect *in vivo* of candidate compounds is one of the preconditions for their use as clinical drugs. Therefore, we evaluated the anti-EV71 effect of LY-55 *in vivo* and found that LY-55 treatment increased the survival rates of EV71-infected mice. Because most of the EV71 infections in patients lead to the spontaneous resolving of symptoms, we also explored the effect of LY-55 against a sub-lethal dose EV71 challenge in mice to imitate the self-limited patients. LY-55 treatment decreased body weight loss (Figure 5B), and the expression of EV71 VP1 protein was significantly decreased in infected mice treated with LY-55. These results showed that LY-55 treatment completely protected the mice from obvious symptoms upon a sub-lethal EV71 challenge.

We found that lycorine derivative LY-55 suppressed EV71 and CVA16 replication at least partly by inhibiting autophagy. Importantly, LY-55 has a good anti-EV71 effect *in vivo*. These findings suggest that LY-55 might be a potential drug for the treatment of HFMD.

## Data Availability

The raw data supporting the conclusions of this manuscript will be made available by the authors, without undue reservation, to any qualified researcher.

## Ethics Statement

This study was carried out in accordance with the recommendations of the National Guidelines for Housing and Care of Laboratory Animals, the Institutional Animal Care and Use Committee. The protocol was approved by the Institutional Animal Care and Use Committee.

## Author Contributions

HW and TG carried out the study and wrote the manuscript. YY synthesized compound LY-55. LY and XP designed the study and manuscript editing. YL designed the study and revised the manuscript. All authors read and approved the final manuscript.

### Conflict of Interest Statement

The authors declare that the research was conducted in the absence of any commercial or financial relationships that could be construed as a potential conflict of interest.
